# Supplementary material for: REM transcription factors and GDE1 shape the DNA methylation landscape through the recruitment of RNA polymerase IV transcription complexes
Source: Nat Cell Biol. 2025 Jun 27;27(7):1136–47. doi: 10.1038/s41556-025-01691-0 (PMC12270911; doi:10.1038/s41556-025-01691-0)
Supplement: Supplementary file 1 — Reporting Summary [file 41556_2025_1691_MOESM1_ESM.pdf]

nature portfolio

Corresponding author(s): Steven E. Jacobsen

Last updated by author(s): Apr 12, 2025

Reporting Summary

Nature Portfolio wishes to improve the reproducibility of the work that we publish. This form provides structure for consistency and transparency in reporting. For further information on Nature Portfolio policies, see our Editorial Policies and the Editorial Policy Checklist.

Statistics

For all statistical analyses, confirm that the following items are present in the figure legend, table legend, main text, or Methods section.

n/a	Confirmed	
☐	☒	The exact sample size (*n*) for each experimental group/condition, given as a discrete number and unit of measurement
☐	☒	A statement on whether measurements were taken from distinct samples or whether the same sample was measured repeatedly
☐	☒	The statistical test(s) used AND whether they are one- or two-sided<br>*Only common tests should be described solely by name; describe more complex techniques in the Methods section.*
☒	☐	A description of all covariates tested
☒	☐	A description of any assumptions or corrections, such as tests of normality and adjustment for multiple comparisons
☐	☒	A full description of the statistical parameters including central tendency (e.g. means) or other basic estimates (e.g. regression coefficient) AND variation (e.g. standard deviation) or associated estimates of uncertainty (e.g. confidence intervals)
☐	☒	For null hypothesis testing, the test statistic (e.g. *F*, *t*, *r*) with confidence intervals, effect sizes, degrees of freedom and *P* value noted<br>*Give P values as exact values whenever suitable.*
☒	☐	For Bayesian analysis, information on the choice of priors and Markov chain Monte Carlo settings
☒	☐	For hierarchical and complex designs, identification of the appropriate level for tests and full reporting of outcomes
☒	☐	Estimates of effect sizes (e.g. Cohen's *d*, Pearson's *r*), indicating how they were calculated

Our web collection on statistics for biologists contains articles on many of the points above.

Software and code

Policy information about availability of computer code

Data collection

No software was used for data collection.

Data analysis

ChIP-seq analysis and DAP-seq analysis: Raw reads were aligned to the Arabidopsis reference genome (TAIR10) with Bowtie2 (v2.3.4.3), allowing only uniquely mapped reads with perfect matches. The Samtools version 1.9 was used to remove duplicated reads. The deeptools version 3.1.3 was used to generate Bigwig tracks. Peaks were called using MACS2 (v2.1.1).

Differentially ChIP-seq localization analysis: ChIP-seq levels at the clsy3 clsy4-dependent siRNA regions were quantified with the HOMER (v4.11.1) annotatePeaks.pl script using the "-noadj, -size given and -len 1" options. Differentially expressed 24nt-siRNA compared to the WT controls were then identified using DESeq (version 1.42.1)(log2 FC≥1 and FDR≤0.05). The data were plotted using the R package ggplot (v3.5.1).

Binding motif analysis: MEME 5.5.0 was used to discover the motifs of the ChIP-seq data sets. FIMO (v5.5.7) was used to scan genome-wide distributions of clsy3 clsy4 motif1 TTTTGCTTAT (single-repeat) with one mismatch allowed, TTTTGCTTATNTTTTGCTTAT (double-repeats) with one mismatch allowed in each repeat, TTTTGCTTATNTTTTGCTTATNTTTTGCTTAT (triple-repeats) with one mismatch allowed in each repeat, and TTTTGCTTATNNTTTTGCTTAT (double-repeats with a two-nucleotide space) with one mismatch allowed in each repeat; clsy3 clsy4 motif2 AAGCGGATNAAGCGGATNAAGCGGAT with p-value less than 5E-09 and q-value less than 0.025. Tomtom (v5.5.7) was used to analyze the similarities between motifs.

WGBS analysis: WGBS raw reads were aligned to both strands of reference genome TAIR10 using BSMAP (v.2.74) with allowing up to 2 mismatches and 1 best hit (-v 2 -w 1). Reads with more than 3 consecutively methylated CHH sites were considered as non-converted reads and removed. Methylation levels were calculated with the ratio of C/(C + T).

RNA-seq analysis: Col-0 leaf, meiocyte and tapetum RNA seq data were downloaded from NCBI Gene Expression Omnibus (GEO) as accession GSM2306324; GSM2306325; GSM2306326, GSM2306313; GSM2306314; GSM2306315 and GSM4911399; GSM4911400; GSM4911401(13), respectively. All raw reads of RNA-seq data were aligned to reference genome TAIR10 by Bowtie2 (v2.3.4.3), and expression abundance was calculated by RSEM (v1.3.1) with default settings. The bamCoverage of deeptools version 3.1.3 was used to normalize the data with RPKM .

Small RNA-seq analysis: Adaptor sequence (TGGAATTCTCGG) of small RNA-seq reads were trimmed with trim_galore, and trimmed reads were mapped to the reference genome TAIR10 using Bowtie2 (v2.3.4.3) with only one unique hit and zero mismatches. sRNA reads that mapped to chloroplast, mitochondrial DNA, tRNA, rRNA, small nucleolar RNAs (snoRNAs), and small nuclear RNAs (snRNAs) were removed using bedtools (v2.26.0). The deeptools version 3.1.3 was used to generate Bigwig tracks. The bamCoverage of deeptools version 3.1.3 was used to normalize the data with RPKM.

Differentially expressed (DE) 24nt-siRNA clusters analysis: Pol IV dependent master siRNA were defined from a previous publication. 24nt-siRNA levels at the master 24nt-siRNA were quantified with the HOMER (v4.11.1) annotatePeaks.pl script using the "-noadj, -size given and -len 1" options. 24nt-siRNA expression levels were normalized by total miRNA amount, which were defined from previously. Differentially expressed 24nt-siRNA compared to the WT controls were then identified using DESeq (version 1.42.1)(log2 FC≤1 and FDR≤0.05).

Quantitative proteomics: Label-free quantification was performed using the MaxQuant software package (v1.6.17.0) with LFQ default setting54, and Arabidopsis TAIR 10 proteome database was used for the database search. Trypsin digestion was applied and a maximum of two missed cleavages were allowed in all searches for tryptic peptides of length 8–40 amino acids. In all, 1% false discovery rate was used as a filter at both protein and peptide-spectrum match (PSM) levels. IP-MS of Col-0 plant tissue was used as control. The empirical Bayes test performed by LIMMA was used for statistical analysis.

For manuscripts utilizing custom algorithms or software that are central to the research but not yet described in published literature, software must be made available to editors and reviewers. We strongly encourage code deposition in a community repository (e.g. GitHub). See the Nature Portfolio guidelines for submitting code & software for further information.

Data

Policy information about availability of data

All manuscripts must include a data availability statement. This statement should provide the following information, where applicable:

- Accession codes, unique identifiers, or web links for publicly available datasets
- A description of any restrictions on data availability
- For clinical datasets or third party data, please ensure that the statement adheres to our policy

All the high-throughput sequencing data generated in this study is accessible at NCBI's Gene Expression Omnibus (GEO) via GEO Series accession number (GSE269181). The mass spectrometry proteomics data generated in this study have been deposited in the ProteomeXchange Consortium via the MassIVE partner repository under accession code MSV000097625. The TAIR10 genome is available at https://www.arabidopsis.org/index.jsp. The Col-0 leaf, meiocyte and tapetum RNA seq data used in this study are available in the National Center for Biotechnology information Gene Expression Omnibus database under accession code GSM2306324; GSM2306325; GSM2306326, GSM2306313; GSM2306314; GSM2306315 and GSM4911399; GSM4911400; GSM4911401, respectively. Source data are provided as a Source Data file.

Research involving human participants, their data, or biological material

Policy information about studies with human participants or human data. See also policy information about sex, gender (identity/presentation), and sexual orientation and race, ethnicity and racism.

Reporting on sex and gender	N.A.
Reporting on race, ethnicity, or other socially relevant groupings	N.A.
Population characteristics	N.A.
Recruitment	N.A.
Ethics oversight	N.A.

Note that full information on the approval of the study protocol must also be provided in the manuscript.

Field-specific reporting

Please select the one below that is the best fit for your research. If you are not sure, read the appropriate sections before making your selection.

☒ Life sciences ☐ Behavioural & social sciences ☐ Ecological, evolutionary & environmental sciences

For a reference copy of the document with all sections, see nature.com/documents/nr-reporting-summary-flat.pdf

Life sciences study design

All studies must disclose on these points even when the disclosure is negative.

Sample size	No sample size calculation was performed. Sample sizes are determined on experimental trials and a previous study (Wang et al., Nature Plants, 2023). Sample sizes of all experiments were large enough (e.g. ovule and anther from >=20 plants were collected for sRNA-seq; unopen buds from >=80 plants were harvested for ChIP-seq with two biological replicates and etc.) to reach statistical reproducibility and significance.
Data exclusions	No data exclusion in the study.
Replication	Two replicates for ChIP-seq and WGBS. Three replicates for sRNA-seq. All replicates were performed independently and produced high reproducible results.
Randomization	For all experiments, treatment and control samples were grown side by side. Allocation of samples were not random, because it is not relevant to the study.
Blinding	No blinding used because it was largely not relevant to our study. All data were collected based on the genotype of plants, while blinding the samples during the experiments will increases the risk of mislabeling and wrong results.

Reporting for specific materials, systems and methods

We require information from authors about some types of materials, experimental systems and methods used in many studies. Here, indicate whether each material, system or method listed is relevant to your study. If you are not sure if a list item applies to your research, read the appropriate section before selecting a response.

Materials & experimental systems

n/a	Involved in the study	
☐	☒	Antibodies
☒	☐	Eukaryotic cell lines
☒	☐	Palaeontology and archaeology
☒	☐	Animals and other organisms
☒	☐	Clinical data
☒	☐	Dual use research of concern
☐	☒	Plants

Methods

n/a	Involved in the study	
☐	☒	ChIP-seq
☒	☐	Flow cytometry
☒	☐	MRI-based neuroimaging

Antibodies

Antibodies used	Antibody for FLAG epitope (for ChIP-seq): M2 antibody, Sigma F1804, 10 ul per ChIP added at a final dilution of 1:400<br>Antibody for MYC epitope (for ChIP-seq): Cell Signaling, 71D10, 20 ul per ChIP added at a final dilution of 1:200<br>HRP conjugated antibody for FLAG epitope (for western blot): Sigma-Aldrich ANTI-FLAG M2-peroxidase A8592, 1:7500 dilution<br>HRP conjugated antibody for MYC epitope (for western blot): Santa Cruz Biotechnology Anti-Myc/c-Myc antibody 9E10 HRP (sc-40 HRP), 1:3000 dilution
Validation	Anti-FLAG M2 (Sigma): the antibodies have been validated by the manufacturer, https://www.sigmaaldrich.com/catalog/product/sigma/fl804<br>Anti-FLAG M2-Peroxidase (HRP)(Sigma): the antibodies have been validated by the manufacturer, https://www.sigmaaldrich.com/US/en/product/sigma/a8592<br>Anti-myc (Cell Signaling): the antibodies have been validated by the manufacturer, https://www.cellsignal.com/products/antibody-conjugates/myc-tag-71d10-rabbit-mab-hrp-conjugate/14038<br>HRP conjugated antibody for MYC epitope (Santa Cruz Biotechnology sc-40): the antibodies have been validated by the manufacturer, https://www.scbt.com/p/c-myc-antibody-9e10?gclid=CjwKCAjwvJyjBhApEiwAWz2nLQpNcYGsOfC7x6jRDD1GtD1Y8eousO7TM84Gg9FKaaHq8gTwQEtgyhoCSMAQAvD_BwE

Dual use research of concern

Policy information about dual use research of concern

Hazards

Could the accidental, deliberate or reckless misuse of agents or technologies generated in the work, or the application of information presented in the manuscript, pose a threat to:

No	Yes	
☒	☐	Public health
☒	☐	National security
☒	☐	Crops and/or livestock
☒	☐	Ecosystems
☒	☐	Any other significant area

Experiments of concern

Does the work involve any of these experiments of concern:

No	Yes	
☒	☐	Demonstrate how to render a vaccine ineffective
☒	☐	Confer resistance to therapeutically useful antibiotics or antiviral agents
☒	☐	Enhance the virulence of a pathogen or render a nonpathogen virulent
☒	☐	Increase transmissibility of a pathogen
☒	☐	Alter the host range of a pathogen
☒	☐	Enable evasion of diagnostic/detection modalities
☒	☐	Enable the weaponization of a biological agent or toxin
☒	☐	Any other potentially harmful combination of experiments and agents

Plants

Seed stocks	Col-0 ecotype was obtained from SALK institute. T-DNA lines used in this study are listed as below: gde1-1 (SALKseq_10069.1), clsy3-1 (SALK_040366), and clsy4-1 (SALK_003876). All if from ABRC.
Novel plant genotypes	rem46 val rem12 triple mutants were generated using guides: aagagttagggtttcaaagagg and TCAAGATTCACATTATCAGTGG. Guides were cloned into pBEE401 vector and the vector was transformed into Col-0, dge1-1, and clsy3 clsy4 muants.<br>rem8-cr plants were generated using guides:tgagcaaggcaaacaaaaATGG and CCTCAGGGGATAAcagagcttt. Guides were cloned into pBEE401 vector and the vector was transformed into Col-0.
Authentication	T-DNA mutants were genotyped by PCR using the primers suggested by SALK (http://signal.salk.edu/tdnaprimers.2.html). CRISPR mutants were Sanger sequenced to confirm the mutations.

ChIP-seq

Data deposition

☒ Confirm that both raw and final processed data have been deposited in a public database such as GEO.

☒ Confirm that you have deposited or provided access to graph files (e.g. BED files) for the called peaks.

Data access links<br>*May remain private before publication.*	All the high-throughput sequencing data generated in this study is accessible at NCBI's Gene Expression Omnibus (GEO) via GEO Series accession number (GSE269181).
Files in database submission	ChIP-seq-Col-9myc-rep1.bw<br>ChIP-seq-Col-9myc-rep2.bw<br>ChIP-seq-CLSY3-9myc-rep1.bw<br>ChIP-seq-CLSY3-9myc-rep2.bw<br>ChIP-seq-CLSY4-9myc-rep1.bw<br>ChIP-seq-CLSY4-9myc-rep2.bw<br>ChIP-seq-CLSY3-9myc-gde1-rep1.bw<br>ChIP-seq-CLSY3-9myc-gde1-rep2.bw<br>ChIP-seq-CLSY3-9myc-rep1.narrowPeak<br>ChIP-seq-CLSY3-9myc-rep2.narrowPeak<br>ChIP-seq-CLSY4-9myc-rep1.narrowPeak<br>ChIP-seq-CLSY4-9myc-rep2.narrowPeak<br>ChIP-seq-CLSY3-9myc-gde1-rep1.narrowPeak<br>ChIP-seq-CLSY3-9myc-gde1-rep2.narrowPeak<br>ChIP-seq-CLSY4-9myc-gde1-rep1.narrowPeak<br>ChIP-seq-CLSY4-9myc-gde1-rep2.narrowPeak<br>ChIP-seq-PolIV-9myc_rep2.narrowPeak<br>ChIP-seq-PolIV-9myc-gde1-rep1.narrowPeak<br>ChIP-seq-PolIV-9myc-gde1-rep2.narrowPeak

ChIP-seq-REM8-9myc_rep1.narrowPeak
ChIP-seq-REM8-9myc_rep2.narrowPeak
ChIP-seq-REM13-9myc-rep1.narrowPeak
ChIP-seq-REM13-9myc-rep2.narrowPeak
ChIP-seq-REM19-9myc-rep1.narrowPeak
ChIP-seq-REM19-9myc-rep2.narrowPeak
ChIP-seq-REM22-9myc-rep1.narrowPeak
ChIP-seq-REM22-9myc-rep2.narrowPeak
ChIP-seq-VAL-9myc-rep1.narrowPeak
ChIP-seq-VAL-9myc-rep2.narrowPeak
ChIP-seq-VDD-9myc-gde1-rep1.narrowPeak
ChIP-seq-VDD-9myc-gde1-rep2.narrowPeak
ChIP-seq-VDD-9myc-rep1.narrowPeak
ChIP-seq-VDD-9myc-rep2.narrowPeak
ChIP-seq-GDE1_3FLAG-rep1.narrowPeak
ChIP-seq-GDE1_3FLAG-rep2.narrowPeak
ChIP-seq-NPVDDZFcol-rep1.narrowPeak
ChIP-seq-CLSY4-9myc-gde1-rep1.bw
ChIP-seq-CLSY4-9myc-gde1-rep2.bw
ChIP-seq-PolIV-9myc_rep1.bw
ChIP-seq-PolIV-9myc_rep2.bw
ChIP-seq-PolIV-9myc-gde1-rep1.bw
ChIP-seq-PolIV-9myc-gde1-rep2.bw
ChIP-seq-REM8-9myc_rep1.bw
ChIP-seq-REM8-9myc_rep2.bw
ChIP-seq-REM13-9myc-rep1.bw
ChIP-seq-REM13-9myc-rep2.bw
ChIP-seq-REM19-9myc-rep1.bw
ChIP-seq-REM19-9myc-rep2.bw
ChIP-seq-REM22-9myc-rep1.bw
ChIP-seq-REM22-9myc-rep2.bw
ChIP-seq-VAL-9myc-rep1.bw
ChIP-seq-VAL-9myc-rep2.bw
ChIP-seq-VDD-9myc-gde1-rep1.bw
ChIP-seq-VDD-9myc-gde1-rep2.bw
ChIP-seq-VDD-9myc-rep1.bw
ChIP-seq-VDD-9myc-rep2.bw
ChIP-seq-Col_3FLAG-rep1.bw
ChIP-seq-Col_3FLAG-rep2.bw
ChIP-seq-GDE1_3FLAG-rep1.bw
ChIP-seq-GDE1_3FLAG-rep2.bw
ChIP-seq-NPVDDZFcol-rep1.bw
ChIP-seq-NPVDDZFcol-rep2.bw
ChIP-seq-VDD-3FLAG-rep1.bw
ChIP-seq-VDD-3FLAG-rep2.bw
ChIP-seq-VDD-3FLAG-rep1-clsy34.bw
ChIP-seq-VDD-3FLAG-rep2-clsy34.bw

Genome browser session
(e.g. UCSC)

Available at GEO

Methodology

Replicates

2

Sequencing depth

ChIP-seq-CLSY3-9myc-gde1-rep1 38322895 22468079 50 PE
ChIP-seq-CLSY3-9myc-gde1-rep2 29940352 17188455 50 PE
ChIP-seq-CLSY3-9myc-rep1 41899996 26416716 50 PE
ChIP-seq-CLSY3-9myc-rep2 29741279 17926402 50 PE
ChIP-seq-CLSY4-9myc-gde1-rep1 39976730 28135934 50 PE
ChIP-seq-CLSY4-9myc-gde1-rep2 24208015 16477456 50 PE
ChIP-seq-CLSY4-9myc-rep1 37634222 24080063 50 PE
ChIP-seq-CLSY4-9myc-rep2 21349871 12871954 50 PE
ChIP-seq-Col-9myc-rep1 29647635 18137933 50 PE
ChIP-seq-Col-9myc-rep2 27979739 18669883 50 PE
ChIP-seq-Col_3FLAG-rep1 43742144 31594808 50 PE
ChIP-seq-Col_3FLAG-rep2 36127929 20249874 50 PE
ChIP-seq-GDE1_3FLAG-rep1 29607543 19960847 50 PE
ChIP-seq-GDE1_3FLAG-rep2 33774325 18050851 50 PE
ChIP-seq-NPVDDZFcol-rep1 64890680 52519488 50 PE
ChIP-seq-NPVDDZFcol-rep2 5925518 4265145 50 PE
ChIP-seq-PolIV-9myc-gde1-rep1 37298313 23347234 50 PE
ChIP-seq-PolIV-9myc-gde1-rep2 20151237 11774044 50 PE
ChIP-seq-PolIV-9myc_rep1 33060928 19661423 50 PE
ChIP-seq-PolIV-9myc_rep2 19737587 11729317 50 PE
ChIP-seq-REM13-9myc-rep1 22759340 12182140 50 PE

ChIP-seq-REM13-9myc-rep2 21816313 11708062 50 PE
ChIP-seq-REM19-9myc-rep1 18032889 9739125 50 PE
ChIP-seq-REM19-9myc-rep2 16623085 8499778 50 PE
ChIP-seq-REM22-9myc-rep1 29047814 17315024 50 PE
ChIP-seq-REM22-9myc-rep2 45598950 35270637 50 PE
ChIP-seq-REM8-9myc_rep1 22709199 11551560 50 PE
ChIP-seq-REM8-9myc_rep2 33970098 13074494 50 PE
ChIP-seq-VAL-9myc-rep1 40593954 24757138 50 PE
ChIP-seq-VAL-9myc-rep2 27496193 17150030 50 PE
ChIP-seq-VDD-3FLAG-rep1-clsy34 81994167 69007097 50 PE
ChIP-seq-VDD-3FLAG-rep1 87821754 74713702 50 PE
ChIP-seq-VDD-3FLAG-rep2-clsy34 8820589 6304238 50 PE
ChIP-seq-VDD-3FLAG-rep2 8883799 6657747 50 PE
ChIP-seq-VDD-9myc-gde1-rep1 43128548 24416059 50 PE
ChIP-seq-VDD-9myc-gde1-rep2 28382558 16987237 50 PE
ChIP-seq-VDD-9myc-rep1 38404573 21434747 50 PE
ChIP-seq-VDD-9myc-rep2 21918605 11938940 50 PE

Antibodies

Anti-FLAG M2 (Sigma) 1:400 dilution
Anti-myc Cell Signaling 1:200 dilution

Peak calling parameters

-g 119146348 -q 0.01 -f BAM

Data quality

All identified peaks in the study were called with a qval threshold of 0.01 (FDR 1%).

Software

Trim Galore (v 0.6.7)
bowtie2 (v 2.3.4.3),
samtools (v 1.9)
MACS2 (v 2.1.1)
deeptools (v 3.1.3).
bedtools (v 2.26.0)
DESeq2 (v 1.42.1)
ggplot2 (v 3.5.1)
HOMER (v4.11.1)
FIMO (v5.5.7)
Tomtom (v5.5.7)
MEME 5.5.0
