# Supplementary figures and images for: REM transcription factors and GDE1 shape the DNA methylation landscape through the recruitment of RNA polymerase IV transcription complexes
Source: Nat Cell Biol. 2025 Jun 27;27(7):1136–47. doi: 10.1038/s41556-025-01691-0 (PMC12270911; doi:10.1038/s41556-025-01691-0)

Extended Data Fig. 2b

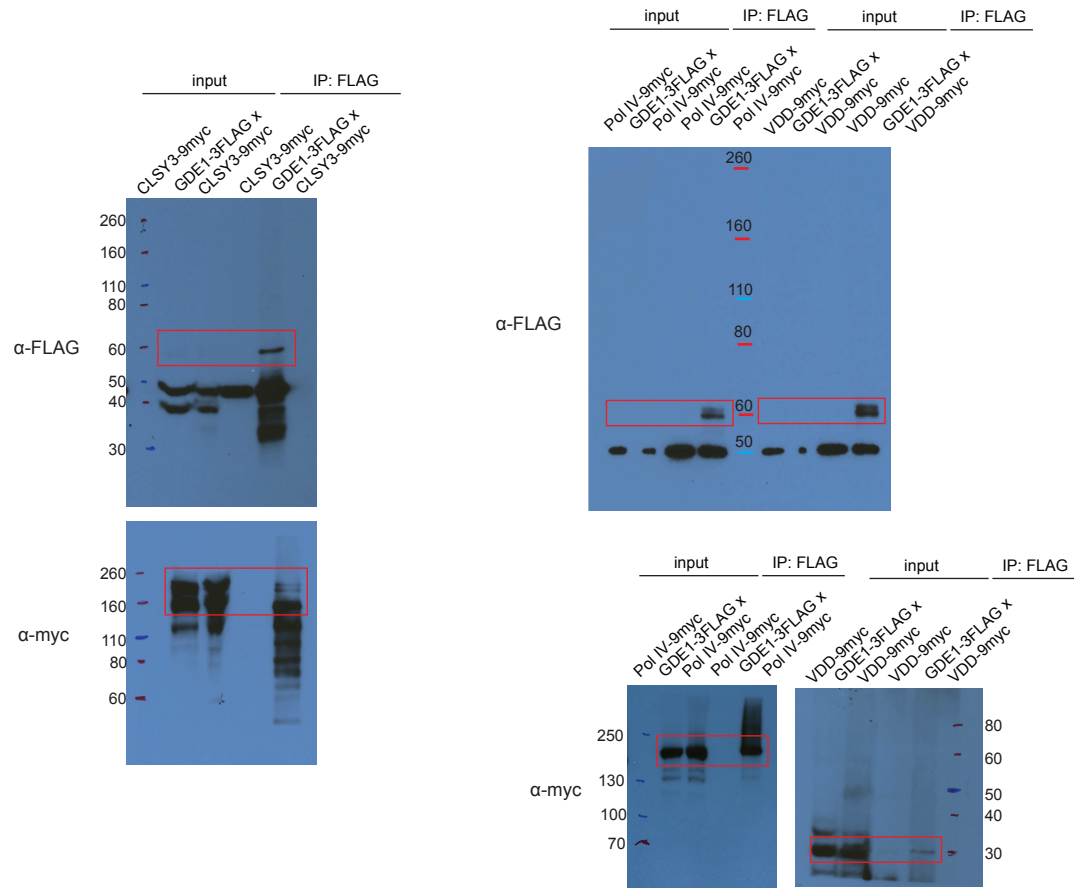

Extended Data Fig. 5a

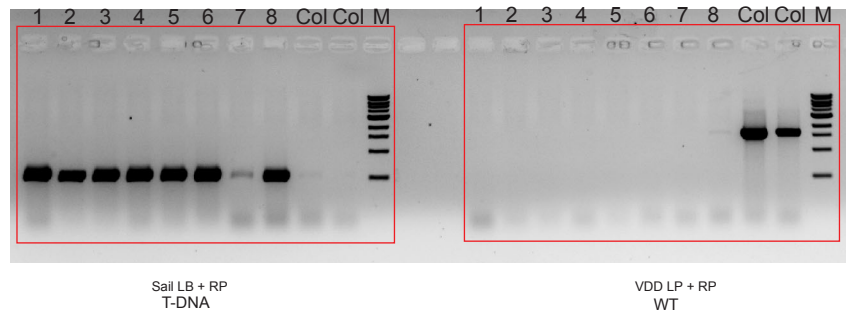

Supplement: Supplementary file 4 — Uncropped western blots. [file 41556_2025_1691_MOESM4_ESM.pdf]
